# Interposition grafting of collagen-gelatin sponge impregnated with basic fibroblast growth factor in primary palatoplasty

**DOI:** 10.1016/j.reth.2023.07.010

**Published:** 2023-08-04

**Authors:** Motoki Katsube, Natsuko Utsunomiya, Yasuhiro Katayama, Hiroki Yamanaka, Itaru Tsuge, Yoshihiro Sowa, Michiharu Sakamoto, Naoki Morimoto

**Affiliations:** Department of Plastic and Reconstructive Surgery, Kyoto University Graduate School of Medicine, 54 Kawahara-cho, Shogoin, Sakyo-ku, Kyoto 606-8507, Japan

**Keywords:** Artificial dermis, Acellular dermal matrix, Palatoplasty, Palatal fistula

## Abstract

**Introduction:**

An oronasal fistula is a challenging post-operative complication of palatoplasty due to impaired velopharyngeal function or its high recurrence rate. Muscle repositioning, a key procedure in palatoplasty, causes dead space at the junction between the hard and soft palates. Consequently, thin oral and nasal mucosae are prone to break down and form fistulas. In this study, we used basic fibroblast growth factor-impregnated collagen gelatin sponge (bFGF-CGS) in primary palatoplasty to reduce fistula formation.

**Methods:**

This retrospective study assessed the complications and efficacy of bFGF-CGS to reduce fistula formation. Patients who underwent primary palatoplasty with bFGF-CGS were included. The same number of patients who underwent primary palatoplasty without bFGF-CGS was included as a control group. The outcomes included post-operative oronasal fistula formation, delayed healing, bleeding, and infection.

**Results:**

Both groups included 44 patients. Except for age at palatoplasty, there were no statistically significant demographic differences between the two groups; however, the rates of fistula formation in the study and control group were 2.3% and 13.6%, respectively. There were no infections among the patients.

**Conclusions:**

The grafting of bFGF-CGS in primary palatoplasty was safe and probably effective in reducing post-operative oronasal fistula formation.

## Highlights

• First report using bFGF-CGS for primary palatoplasty
• The grafting of bFGF-CGS for palatoplasty was safe
• The grafting of bFGF-CGS reduced the post-operative fistula formation

## 1 Introduction

Palatoplasty is a surgical procedure for separating the oral and nasal cavities to improve velopharyngeal function (VPF). Although various surgical techniques have been developed to improve VPF and ensure safe closure, some complications, including post-operative fistula formation, are not uncommon.

In patients with cleft palate, the levator veli palatini and palato-pharyngeus muscles, which play a key role in VPF, are abnormally oriented in an anteroposterior direction and inserted into the posterior edge of the hard palate [1]. In 1969, Kriens [2] advocated for the posterior repositioning of the levator veli palatini and palato-pharyngeus muscles, an operation known as intra-velar veloplasty (IVV). Sommerlad [3] encouraged the use of a surgical microscope to perform a more accurate IVV procedure, and Andrades et al. [4] suggested practicing a more radical IVV, reporting favorable effects on speech outcomes. Furlow [5] recommended opposing the Z-plasty of the oral and nasal mucosas, as well as muscle reconstruction. In any case, the most crucial procedure in this technique is the repositioning of muscles in the posterior region.

Oronasal fistula formation after palatoplasty is one of the major concerns since it potentially compromises oronasal function and has a high recurrence rate [6,7]. A fistula cause food or fluid to escape into the nose, nasal air leakage, or speech distortion. Hardwicke et al. [8] reviewed 9,294 patients who underwent primary palatoplasty, reporting an overall fistula formation incidence of 8.4%. After posterior positioning of the palatal muscles, the soft tissues around the region are very thin and prone to breakdown. The incidence of post-operative fistulas is highest at the junction of the hard and soft palates, where the cleft width and the amount of repair stress are highest [9,10]. In this sense, fistula formation in the oronasal region has been attributed to several causes, including wound disruption caused by tension on the closure wound, infection, flap trauma, and hematoma [11]. Fistula closure is difficult due to underlying fibrosis around the fistula, insufficient soft tissue, and insufficient mucous mobility. Various procedures have been developed to avoid fistula formation, including the application of a buccal fat flap, autologous dermis graft, or acellular dermal matrix (ADM) [[12], [13], [14], [15], [16], [17], [18], [19], [20], [21], [22], [23]]. Several clinical studies have used ADM for primary palatoplasty since an initial report by Clark et al. [24] in 2003. ADM has been reported to be effective in reducing palatal fistula formation after primary palatoplasty [7,17,25,26] and is currently being used in clinical procedures worldwide [16]. Morimoto et al. [27] developed a novel hybrid ADM, which includes a drug delivery system that consists of a basic fibroblast growth factor (bFGF) composed of collagen-gelatin sponge (CGS). In Japan, bFGF is widely used to promote granulation and angiogenesis in skin ulcers and burn wounds. A CGS impregnated with bFGF (bFGF-CGS) consists of 10% alkali-treated gelatin, which binds to positively charged bFGF during biodegradation, gradually releasing the bFGF [28]. This procedure has been proven highly effective in epithelialization, granulation, and vascularization in wound-healing [26,29,30].

We used the bFGF-CGS as an interpositional graft at the junction of the hard and soft palates after repositioning the muscles during primary palatoplasty. This study aimed to evaluate the risk of using bFGF-CGS, including infection, delayed healing, or bleeding, and the efficacy of our procedure for oronasal fistula formation.

## 2 Methods

### 2.1 Study design

We conducted a retrospective review of consecutive primary palatoplasty cases performed at our institution. From March 2020 to December 2022, we performed interposition grafting of a 9 cm^2^ CGS without an upper silicone sheet (PELNAC Gplus®, Gunze Co., Ltd., Ayabe, Japan) impregnated with 125 μg bFGF (Fiblast® Spray; Kaken Pharmaceutical Co., Ltd., Tokyo, Japan) (bFGF-CGS) at the junction of the hard and soft palates after muscle repositioning in primary palatoplasty using the radical IVV or Furlow technique. All patients who had primary palatoplasty performed during the study period underwent this procedure and were included in the study group. Before March 2020, no patients underwent this procedure. The control group consisted of a consecutive and equal number of patients who underwent primary palatoplasty without the use of bFGF-CGS between March 2017 and January 2020.

This study received IRB approval from the Kyoto University Ethics Committee (R3528). The study was conducted following the Declaration of Helsinki guidelines.

### 2.2 Data collection

Demographic data from the patients were corrected for age at the time of palatoplasty, gender, associated diseases, type of cleft palate according to the Veau classification, cleft palate width, posterior arch width, ratio of cleft width to the posterior arch width, and soft palate surgical characteristics. Regarding the post-operative conditions, medical records were reviewed, including oronasal fistula formation, delayed healing, bleeding, and infections. Furthermore, oronasal fistula formation details were reviewed, including age at palatoplasty, gender, type of cleft palate according to the Veau classification, and location of oronasal fistula according to the Pittsburgh fistula classification.

The Veau classification includes four types of cleft palate: clefts of the soft palate (type I), cleft of the hard and soft palates (type II), unilateral cleft of alveolus and palate (type III), and bilateral cleft of alveolus and palate (type IV). The cleft palate and posterior arch widths were measured as the distance between the cleft edges at the intertuberosity region and the distance between the two maxillary tuberosities, according to Parwaz et al. [31] These parameters were measured at the beginning of the surgery, before the local anesthesia injection. Delayed healing was defined as oral mucosal dehiscence or ulcer requiring more than two weeks to heal. Post-operative infection was defined as redness, swelling, or discharge at the site of surgery, requiring antibiotics. Post-operative fistula formation was defined as a tunnel from the oral cavity to the nasal cavity due to a breakdown or failure of palatal repair. The location of the post-operative oronasal fistula was defined according to the Pittsburgh fistula classification. The classification includes seven fistula types: fistulas at the uvula (type I), within the soft palate (type II), at the junction of the hard and soft palates (type III), within the hard palate (type IV), at the incisive foramen or junction of the primary and secondary palates (type V), lingual to alveolar (type VI), and labial-alveolar (type VII) [32].

### 2.3 Surgical procedure

**Fig. 1:**
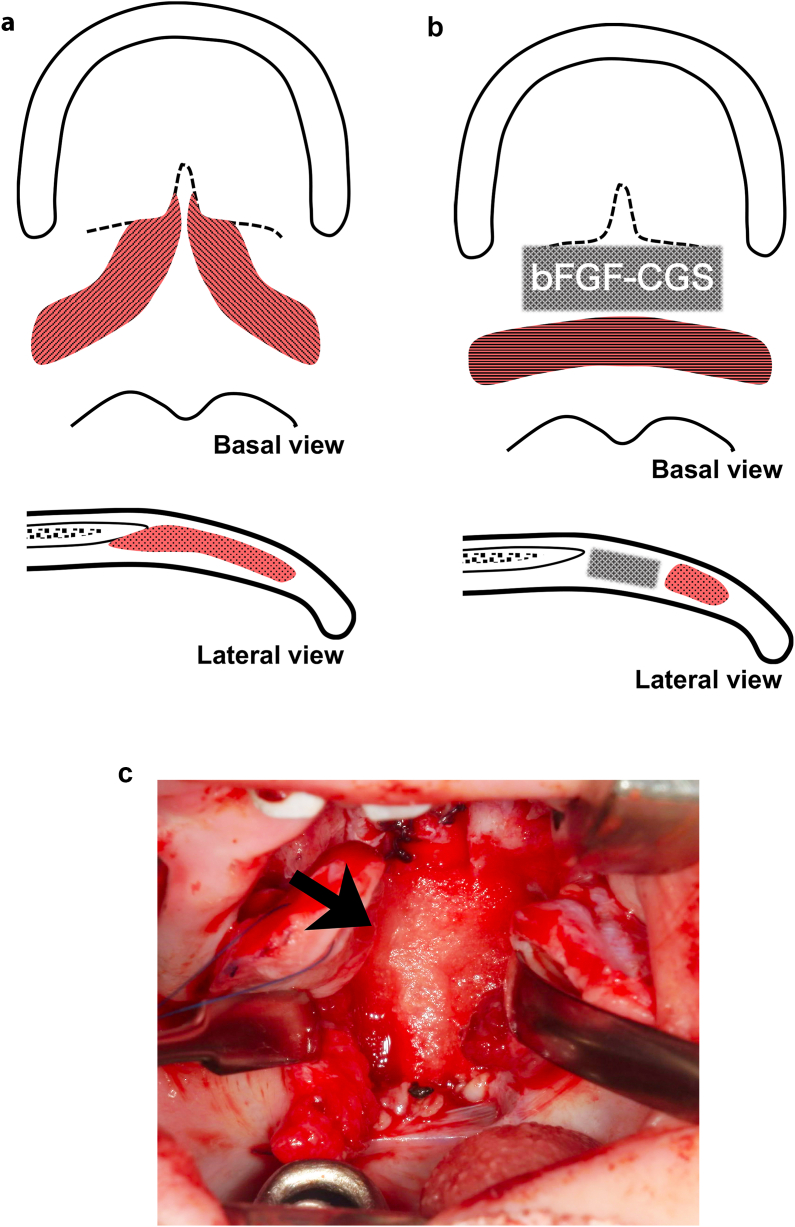
BFGF-CGS utilization in palatoplasty. (a) The levator veli palatini muscle and palato-pharyngeus muscle in a patient with cleft palate are abnormally oriented antero-posteiror direction and inserted into the posterior edge of the hard palate. (b) After muscle repositioning, bFGF-CGS was used as interposition grafting at the junction of the hard and soft palates. (c) Clinical picture after the grafting, with a black arrow indicating bFGF-CGS.

Abnormally oriented muscles were repaired using radical IVV or the Furlow technique with a surgical microscope. After complete muscle dissection and mobilization, the nasal mucosa was closed with absorbable sutures and the muscles were overlapped and sutured. Hard palate closure was performed using relaxing incisions (von Langenbeck or Bardach styles). In the study group, patients underwent the following procedure: a bFGF-CGS was trimmed to fit the dead space at the junction of the hard and soft palates and placed there without any fixation (Fig. 1). Finally, the soft palate oral mucosa was closed with absorbable sutures in both groups. A single surgeon performed all surgeries in the study group, whereas three surgeons performed the surgeries in the control group. All the surgeons were experts in the field of facial surgery.

### 2.4 Statistical analysis

The demographic differences between the two groups were statistically compared using the *t*-test, the Chi-square test, or the Fisher exact test (R 4.0.0, R Core Team, 2020). The statistical significance was set at *P* < 0.05.

## 3 Results

Patient demographics are listed in Table 1. Both groups included 44 patients. Except for the age at palatoplasty (*P* = 0.0439), no statistical significance was found in the demographics data. In the study group, the mean palatoplasty age was 22.3 months, with a median of 13 months (range: 9–151 months) and a standard deviation (SD) of 24 months. On the other hand, in the control group, the mean palatoplasty age was 14.6 months, with a median of 13 months (range: 10–33 months) and an SD of 4.0 months. The study group had 19 males and 25 females, whereas the control group had 23 males and 21 females. This difference was not statistically significant (*P* = 0.5223). The associated diseases in the study group were as follows: two patients with Wolf-Hirshhorn syndrome, three with Pierre Robin syndrome, two with trisomy 21, one with trisomy 13, one with 22q.11.2 deletion syndrome, one with West syndrome, and one with a diaphragmatic hernia and lung hypoplasia. The associated diseases in the control group included two patients with CHARGE syndrome, one with 18q deletion syndrome, and one with corrected transposition of the great arteries. The difference between the two groups was not statistically significant (*P* = 0.2288). In the study group, the age at palatoplasty was higher in 10 patients due to associated disease and in four patients due to late initial consultation. In the control group, this age was higher in four patients due to associated disease and in one patient due to late initial consultation in the control group. Excluding these patients, in the study group the mean age at the palatoplasty was 12.6 months, with a median of 12 months (range: 9–25 months) and an SD of 2.7 months. In contrast, in the control group, the mean age of palatoplasty was 13.6 months, with a median age of 13 months (range: 10–18 months), and an SD of 2.3 months. The difference between the two groups was not statistically significant (*P* = 0.1017). Regarding the Veau classification, the study group presented nine type I, 18 types II, 12 type III, and five type IV, while the control group comprised 14 types I, seven type II, 16 type III, and seven type IV, with no statistically significant differences between the two groups (*P* = 0.0775). In the study group, the cleft palate width, according to Veau classification type II, III, and IV had a mean distance of 9.4 mm, a median of 9 mm (range: 5–15 mm), and an SD of 2.7 mm. The data were not applicable in the control group. In the study group, the posterior arch width had a mean distance of 27.9 mm, a median distance of 28 mm (range: 24–34 mm), and an SD of 2.6 mm. The data were not available in the control group. In the study group, the ratio of cleft width to posterior arch width was 0.33, with a median of 0.33 (range: 0.19–0.52), and an SD of 0.08, while the data were not applicable in the control group. Regarding the surgical characteristics of the soft palate in the study group, 31 and 13 patients underwent IVV and Furlow, respectively, whereas 37 and 7 patients in the control group underwent IVV and Furlow, respectively. This difference was not statistically significant (*P* = 0.2028).

**Table 1:** Demographics.

	Study group	Control group	*P* value
No.	44	44	
Age at palatoplasty (mo), mean (SD)	22.3 ± 24	14.6 ± 4.0	0.0439^a^
Gender			
Male	19	23	0.5223
Female	25	21	
Associated syndromes, n (%)	9 (20.5)	4 (9.1)	0.2288
Mean cleft palate width ± SD, mm	9.4 ± 2.7	N/A	
Posterior arch width	27.9 ± 2.6	N/A	
Ratio of cleft width to the posterior arch width	0.33 ± 0.08	N/A	
Veau classification			0.0775
Type I	9	14	
Type II	18	7	
Type III	12	16	
Type IV	5	7	
Surgery characteristics			0.2028
Intra-velar veloplasty	31	37	
Furlow	13	7	

mo, months; N/A, not available; SD, standard deviation.

a Statistically significant.

The post-operative complications are presented in Table 2. The post-operative oronasal fistula formation occurred in one patient in the study group (2.3%) and in six patients in the control group (13.6%), with no statistically significant differences between the two groups (*P* = 0.1099). One female in the study group and six males in the control group presented fistula formation, respectively. Fistula formation, according to the Veau classification, was as follows: one type I in the study group, and one type I, one type II, three type III, and one type IV in the control group. According to Pittsburgh classification, fistulas were located as follows: one type III in the study group, and one type I, four type III, and one type V in the control group (Table 3). Delayed healing was observed in two patients in the study group and in six in the control group. This difference was not statistically significant (*P* = 0.3524). All of them were closed naturally within four weeks of palatoplasty. None of the patients in the study group had exposure of the graft. Each group had one patient with post-operative bleeding. This difference was not statistically significant (*P* = 0.2288). No post-operative infections were observed.

**Table 2:** Complications.

Complications	Study group	Control group	*P* value
Fistula, n (%)	1 (2.3)	6 (13.6)	0.1099
Delayed healing, n (%)	2 (4.5)	6 (13.6)	0.3524
Bleeding, n (%)	1 (2.3)	1 (2.3)	0.2288
Infection, n (%)	0	0	1

**Table 3:** Comparison of fistula patients between groups.

	Study group	Control group
Occurrence n, (%)	1 (2.3)	6 (13.6)
Mean age ± SD, months	11	13.3 ± 1.75
Gender		
Male	0	6
Female	1	0
Veau classification		
Type I	1	1
Type II	0	1
Type III	0	3
Type IV	0	1
Pittsburgh fistula classification		
Type I	0	1
Type II	0	0
Type III	1	4
Type IV	0	0
Type V	0	1
Type VI	0	0
Type VII	0	0

SD, standard deviation.

## 4 Discussion

To the best of our knowledge, this is the first study to assess the utility of interposition grafting at the junction of the hard and soft palates after muscle repositioning with bFGF-CGS. The use of artificial materials in the oral cavity, such as ADM, raises concerns regarding their negative effects on wound healing. Clark et al. [24] first reported the utility of AlloDerm (LifeCell Corporation, Branchburg, NJ, USA) in primary palatoplasty, concluding that the procedure was safe and effective. Gilardino et al. [17] performed a prospective study of 65 patients with ADM (DermaMatrix, Synthes CMF, West Chester, Pa., USA) and 65 patients without ADM who underwent primary palatoplasty, finding no post-operative infections in either group. Other studies have also found that using ADM in palatoplasty did not have a negative effect on the wound-healing process [22,23,33,34]. In this study, even though several patients presented delayed healing and one patient experienced bleeding post-operatively, the incidence of these complications was lower than that in the control group. Moreover, no post-operative infections were observed in the 44 patients of the study. Therefore, the utilization of bFGF-CGS in primary palatoplasty was demonstrated as safe.

Oronasal fistula formation after palatoplasty can be attributed to many factors, such as the surgeon's skill, age at operation, type of cleft palate, associated syndromes, cleft region width, and dead space formation [35]. In this study, although all surgeons were sufficiently experienced in palatoplasty, they differed between groups. Some studies mentioned that the surgeon's previous experience in palatoplasty was an important factor for palatal fistula formation after surgery [36,37]. However, other studies did not find an association between the surgeon's skills and fistula formation [31,38,39]. In this sense, no consensus has been established on the main cause of fistula formation. Nevertheless, many reports have found that the cleft width is related to fistula occurrence [38]. Some researchers have found that a cleft width equal to or over 15 mm is a risk factor for fistula formation [24,31]. Parwaz et al. [31] found that the ratio of cleft width to posterior arch width was a strong predictor of fistula formation if the ratio was superior to 0.41. In this study, seven patients from the study group with a ratio of 0.41 did not have oronasal fistula or delayed healing (Supplementary material).

Complete muscle repositioning in palatoplasty causes dead space at the junction of the hard and soft palates. To avoid fistula formation at this location, various procedures have been developed, including the application of a buccal fat flap, autologous dermis graft, or ADM [13,23]. Qui et al. [12] performed Furlow palatoplasty with prophylactic augmentation of the buccal fat flap at the junction of the hard and soft palates, reporting its efficacy in improving oral mucosal healing. Khan et al. [13] performed autologous dermal graft placement in primary palatoplasty in 16 patients, finding two post-operative fistula formations (12.5%). Similar to our study, Gilardino et al. [17] placed the ADM at the site of muscle repair. They reported that fistula formation was 1.5% in the ADM group and 12.3% in the control group, concluding that the routine application of ADM reduced fistula formation. In this study, although the statistical difference in fistula formation between the groups was not significant (*P* = 0.1099), the group difference in the incidence of fistulas including all locations was large (2.3% in the study group and 13.6% in the control group). This difference was still present in terms of Pittsburgh fistula classification type III, the location of the bFGF-CGS grafting, with one patient in the study group and four patients in the control group (Table 3). Although the cleft width in the control group was not available, the oronasal fistula formation rate was different in this study, suggesting that the implantation of bFGF-CGS could have an inhibitory effect on fistula development after palatoplasty. Based on previous reports and our study, although it is unclear how to prevent palatal fistula formation during the early stages post-operatively, ADM implantation in palatoplasty at the junction of the hard and soft palates may be effective in reducing the risk of developing palatal fistulas [17,21,[23], [24], [25]].

Various ADMs have been developed and used clinically worldwide [16], although only a few are allowed to be used in Japan. Among these, bFGF-CGS can promote capillary development, granulation tissue generation, and epithelialization [40]. CGS impregnated with 7–14 μg/cm^2^ was the optimal density [26,29,30]. In the present study, the concentration was adjusted for each case.

Although muscle reconstruction in the posterior position is a key component of palatoplasty, there remains the concern that over time, post-operative scar contracture at the edge of the hard and soft palates could cause palatal shortening and deterioration of the VPF. Kirshner et al. [19] performed an animal study on oronasal fistula repair using ADM. They found that ADM induced tissue regeneration without scar formation. Reagan et al. [41] showed that ADM was able to reduce wound contraction in a porcine model. Kakudo et al. [42] implanted subcutaneous CGS, with or without bFGF, into the thoracic region of mice, observing subcutaneous tissue regeneration. They found that, although the CGS without FGF group had no fat pad, the CGS with FGF group had thick fat tissues, concluding that bFGF-CGS was able to enhance adipogenic effects in subcutis. Hence, our procedure could potentially be effective in preventing soft palate shortening and long-term VPF maintenance.

This study has two limitations. First, the surgeons who performed the palatoplasty differed between the groups. Second, the cleft palate width measure was not used in the control group. Both these factors may have influenced the fistula formation rate in this study.

## 5 Conclusion

Interposition grafting of bFGF-CGS following muscle repositioning in palatoplasty is a safe procedure and could be effective in reducing post-operative oronasal fistula formation.

## Declaration of competing interest

None.
